# Supplementary material for: Navigating Professional Identity and Cultural Expectations: A Phenomenological Study of Female Saudi Nurses’ Experiences in Mixed-Gender Healthcare Settings
Source: Healthcare (Basel). 2025 Nov 25;13(23):3042. doi: 10.3390/healthcare13233042 (PMC12692420; doi:10.3390/healthcare13233042)
Supplement: Supplementary file 1 [file healthcare-13-03042-s001.zip › healthcare-3996356-supplementary.pdf]

File S1: THE INTERVIEW GUIDE: MAIN INTERVIEW QUESTIONS
Domain 1: Daily Work Experiences in Mixed-Gender Settings
Opening Question:

"Can you describe a typical workday for you as a nurse in your current facility?"

Probes:

- Morning routine at work
- Typical interactions during the shift
- Most routine or familiar aspects of the day

Transition Question:

"Your facility has both male and female healthcare professionals and patients. How would you describe the general environment where you work?"

Probes:

- Initial feelings working in a mixed-gender environment
- Changes in comfort level over time

Domain 2: Cultural Identity and Professional Role Intersection
Main Questions:

1. "How do you experience the intersection between your identity as a Saudi woman and your professional role as a nurse?"
   Probes: Moments of harmony or tension; meaning-making processes.
2. "What does it mean to you to maintain your Islamic values and Saudi cultural identity while working as a nurse?"
   Probes: Practicing faith at work; balancing religious obligations with clinical demands; important cultural traditions.
3. "Some people might see mixed-gender workplaces as conflicting with traditional Saudi values. How do you view this?"
   Probes: Family responses; personal views; explaining work to others.

Domain 3: Gender Dynamics and Professional Interactions
Main Questions:

1. "Tell me about your experiences working with male healthcare colleagues—doctors, nurses, technicians, or others."
   Probes: Positive interactions; challenging situations; strategies for professional boundaries; evolution over career.
2. "What is it like for you to provide nursing care to male patients?"
   Probes: Specific situations; clinical and cultural considerations; patient/family responses.
3. "Have you ever experienced situations where your professional authority or clinical judgment was questioned because of your gender or cultural background?"
   Probes: Detailed examples; responses; coping strategies.

Domain 4: Professional Identity Development
Main Questions:

1. "How would you describe your professional identity as a nurse? Has it changed since you began your career?"
   Probes: Meaning of being a professional nurse; alignment with Saudi identity; key shaping experiences.
2. "Some nurses talk about feeling like they must 'choose' between their cultural identity and professional identity. What is your experience?"
   Probes: Integration vs. separation of identities; strategies for balancing roles; advice for younger nurses.
3. "Do you see yourself as a role model for other Saudi women in healthcare? Why or why not?"
   Probes: Responsibilities; mentoring; hopes for the next generation.

Domain 5: Organizational Support and Work Environment
Main Questions:

1. "What kind of support—or lack of support—have you experienced from your healthcare organization regarding cultural or gender-related needs?"
   Probes: Helpful policies; accommodations for prayer/holidays; areas needing improvement.
2. "How does your work environment affect your confidence and comfort as a Saudi female nurse?"
   Probes: Feeling valued; stress factors; leadership influence.
3. "Tell me about your relationships with other nurses—both Saudi women and colleagues from other backgrounds."
   Probes: Sources of support; professional friendships; peer support role.

Domain 6: Future Aspirations and Reflections
Closing Questions:

1. "Looking ahead, what are your hopes and goals for your nursing career?"
   Probes: Professional development; working in mixed-gender settings; necessary changes for support.
2. "If you could speak to healthcare leaders or policymakers about the experiences of Saudi female nurses, what would you want them to know?"
   Probes: Suggested improvements; support for cultural integration and professional growth.
3. "Is there anything about your experience that we haven't discussed but that you feel is important?"
   Probes: Additional stories; capturing overall journey.

CLOSING PROTOCOL
Debriefing:

"Thank you for sharing your experiences. How are you feeling after this conversation? Is there anything you'd like to discuss further?"

Member Checking Invitation:

"Would you be willing to review a short summary of the preliminary findings to ensure they reflect your experiences? This would take about 15–20 minutes."

Follow-Up Contact:

"If you think of anything else after today, or have questions, contact me at [contact information]. You will also receive a copy of the final study results if you indicated interest."

Gratitude:

"Your participation makes a meaningful difference. Thank you again for your time and openness."
